# PW02-039 - Long-term anakinra treatment in CAPS: a metaanalys

**DOI:** 10.1186/1546-0096-11-S1-A180

**Published:** 2013-11-08

**Authors:** M Leinonen, B Hallén, M Aldén-Raboisson, H Olivecrona

**Affiliations:** 1 SWEDISH ORPHAN BIOVITRUM, Stockholm, Sweden

## Introduction

The common denominator in CAPS (FCAS, Muckle-Wells syndrome, NOMID/CINCA) is an uncontrolled IL-1β release. An often complete response after treatment with the IL-1 blocker anakinra (Kineret®) has been demonstrated in all three entities of CAPS [1-3]. However, the overall documentation is limited due to the inherent difficulties in conducting randomized studies in the more severe forms of the disease, and the low prevalence of CAPS (1 in 1 000 000). The literature consists of uncontrolled, small clinical studies, and a large number of case reports.

## Objectives

To estimate CAPS disease activity before and after long-term treatment with anakinra by a meta-analysis.

## Methods

Five major data bases were searched to find published data on anakinra in the treatment of CAPS. To be included in the meta-analysis studies had to be prospectively designed and include longitudinal long-term data (at least 6 month follow-up) on the selected endpoints and published in a peer reviewed journal. Presence of primary disease symptoms (rash, headache, arthralgia, and fever) and on levels of inflammation markers (CRP, SAA) were selected as endpoints. Pooled estimates were calculated as weighted average of the individual studies, using the inverse of variability as weights. The main meta-analysis was supported by a sensitivity analysis, conducted by including studies of retrospective nature and/or providing only short-term data. Data from 5 untreated patients were used as a control group [4].

## Results

The search resulted in 14 clinical studies and a large number of published case reports (n=79) on anakinra treatment in all CAPS subtypes. Three studies fulfilled the inclusion studies for the main analysis and three additional studies for the sensitivity analysis. Selected studies comprised both pediatric and adult patients in all three CAPS entities. All four disease symptoms were present in a large proportion of patients at baseline, rash being most frequently (95% CI from 83.5% to 97.5%) and fever least frequently reported (95% CI from 46.6% to 71.8%). At the last follow-up visit (11-60 months) the estimated proportion of affected patients was <20% for each symptom. Almost all patients had abnormal SAA (95% CI from 85.0 to 98.9% of patients) and CRP (95% CI from 91.5% to 100.0%) at baseline. Mean SAA decreased from a baseline value of 41.0 mg/L to 6.9 mg/L at the last visit and CRP from 28.8 to 6.4 mg/L. Among the 5 untreated control patients (NOMID/CINCA), all symptoms except self-reported fever were still present at the last visit at follow-up (median 52 months). The sensitivity analyses showed comparable findings.

## Conclusion

The results of the present meta-analysis of long-term efficacy measured as presence of primary disease symptoms (rash, headache, arthralgia, and fever) and levels of inflammation markers (CRP, SAA), confirm the efficacy of anakinra in the treatment of CAPS, including NOMID, MWS, and FCAS.

## Competing interests

M. Leinonen Consultant for: Swedish Orphan Biovitrum AB, B. Hallén Employee of: Swedish Orphan Biovitrum AB, M. Aldén-Raboisson Employee of: Swedish Orphan Biovitrum AB, H. Olivecrona Employee of: Swedish Orphan Biovitrum AB
